# Milk Protein Glycation Compromises Postprandial Lysine Bioavailability but does not Modulate Postprandial Muscle Protein Synthesis Rates *In Vivo* in Males: A Double-blind, Randomized Parallel Trial^☆^

**DOI:** 10.1016/j.tjnut.2025.05.032

**Published:** 2025-05-27

**Authors:** Glenn AA van Lieshout, Jorn Trommelen, Floris K Hendriks, Jean Nyakayiru, Janneau van Kranenburg, Joan M Senden, Joy PB Goessens, Lex B Verdijk, Marjolijn CE Bragt, Luc JC van Loon

**Affiliations:** 1 Department of Human Biology, NUTRIM Institute of Nutrition and Translational Research in Metabolism, Maastricht University Medical Centre+, Maastricht, the Netherlands; 2 FrieslandCampina, Amersfoort, the Netherlands

**Keywords:** protein digestibility, protein quality, protein supplement, Maillard reaction, muscle recovery, dairy

## Abstract

**Background:**

Industrial processing and storage of milk products can strongly increase protein glycation level. Previously, we have reported that a high protein glycation level impairs protein digestion, thereby compromising lysine bioavailability. The lower postprandial lysine availability may restrict the anabolic properties of a high glycated protein.

**Objectives:**

The objective of this study was to assess the impact of milk protein glycation on postprandial plasma amino acid availability and subsequent postprandial muscle protein synthesis rates during recovery from a single bout of resistance-type exercise.

**Methods:**

Forty-five recreationally active, healthy young males participated in this double-blinded, randomized parallel study. After performing a single bout of whole-body resistance-type exercise, subjects ingested 20 g milk protein with either a low (4%; LOW-GLYC) or high (47%; HIGH-GLYC) glycation level or a noncaloric placebo (PLA). Continuous intravenous infusion of L-[*ring*-^13^C_6_]-phenylalanine was combined with the collection of blood and muscle tissue samples during a 6-h postprandial period to assess plasma amino acid concentrations and muscle protein synthesis rates.

**Results:**

Protein ingestion increased plasma total and essential amino acid concentrations compared with placebo (time × treatment interaction: *P* < 0.001), with no differences between the low and high glycated milk protein. Plasma lysine availability, assessed over the full 6 h postprandial period, was substantially lower following ingestion of the protein with the high versus low glycation level (−5 ± 7 compared with 10 ± 9 mmol · L^-1^ · 360 min, respectively, *P* < 0.001). Postprandial muscle protein synthesis rates did not differ between treatments (0.059 ± 0.016, 0.061 ± 0.012, and 0.061 ± 0.018 % · h^-1^, in LOW-GLYC, HIGH-GLYC and PLA, respectively, *P* = 0.939).

**Conclusions:**

Ingestion of protein with a higher glycation level attenuates postprandial plasma lysine availability. Milk protein glycation does not modulate postprandial muscle protein synthesis rates during recovery from resistance exercise in healthy, young males.

This trial was registered at the Dutch Trial Register as NL8690; https://onderzoekmetmensen.nl/nl/trial/49398.

## Introduction

Muscle is a highly adaptive organ that is constantly turning over, i.e., muscle proteins are continually synthesized and degraded. This turnover allows muscle tissue to adapt to challenges such as exercise. A single session of exercise stimulates muscle protein synthesis rates and, to a lesser extent, muscle protein breakdown rates [1,2]. However, muscle protein net balance will remain negative in the absence of food intake [2]. Many, but not all studies show that protein ingestion stimulates postexercise muscle protein synthesis rates [[3], [4], [5], [6], [7], [8], [9]]. Conversely, protein ingestion inhibits postexercise muscle protein breakdown rates [10]. Together, protein ingestion results in net muscle protein accretion during the acute stages of postexercise recovery [10]. Therefore, postexercise protein ingestion is a widely applied strategy to further augment postexercise muscle protein synthesis rates. The leucine content of a meal has been reported to be an important determinant of the muscle protein synthetic response [[11], [12], [13]]. In addition, an adequate availability of the other essential amino acids (EAAs) is required to allow a more sustained muscle protein synthetic response [14]. This aligns with the concept that the lowest availability of an EAA relative to its requirements represents the limiting factor to support protein synthesis, as endorsed by the WHO [15,16]. The postprandial availability of EAAs following protein ingestion is modulated by the type, amino acid composition, digestion and absorption kinetics, and industrial processing of the protein source [8].

Industrial processing of protein can modulate various characteristics that affect the protein’s digestion and amino acid absorption kinetics [[17], [18], [19]]. For example, hydrolyzed casein is more rapidly digested and absorbed when compared with intact protein [18]. Heat processing and storage of milk proteins have also been shown to strongly affect protein characteristics. Spray drying of milk and high temperature storage of milk powder induces the Maillard reaction [20,21]. Specifically, reducing sugars such as lactose react with free amino groups within the protein, a reaction that is termed protein glycation [22]. Lysine is the amino acid most prone to glycation as its free NH_2_-group makes it particularly vulnerable to glycation [22]. Therefore, the protein glycation level is often expressed as the percentage of blocked lysine, i.e., lysine that is not metabolically available [23]. *In vitro* and *in vivo* animal studies suggest that an elevated protein glycation level results in a decreased protein digestibility [24]. In humans, it has been observed that a higher glycation level in milk protein results in a lower metabolic availability according to the indicator amino acid oxidation technique [25]. In line, we have shown that a high glycation level reduces postprandial plasma EAA availability *in vivo* in humans [17]. This effect was fully attributed to an attenuated postprandial rise in plasma lysine concentrations. In accordance, we recently applied specifically produced intrinsically labeled protein to prove that protein glycation compromises the postprandial bioavailability of milk protein-derived lysine *in vivo* in humans [26]. We speculated that the lower postprandial availability of lysine following ingestion of high glycated protein might impact the biofunctionality of the protein.

Many dairy products contain a considerable level of protein glycation, such as (long-stored) milk powder, infant formula, and dairy protein, based supplements [23,[27], [28], [29], [30]]. In particular, products with a high protein content that are used to support muscle protein synthesis during recovery from exercise, i.e., sports supplements, have been shown to contain glycation levels up to 44% blocked lysine [29,30]. This is attributed to heat treatment during processing, presence of carbohydrates in the product (e.g., weight gainers), and/or the long shelf life of these products [29,30]. A high protein glycation level in sports supplements can restrict postprandial plasma lysine availability, which may compromise postexercise recovery. Hence, we hypothesize that ingestion of high glycated milk protein attenuates postprandial plasma lysine availability compared with a low glycated milk protein, resulting in lower muscle protein synthesis rates during recovery from a bout of resistance-type exercise.

In the present study, we recruited 45 healthy young males to assess the impact of milk protein glycation level on postprandial plasma amino acid availability, whole-body protein metabolism, and muscle protein synthesis rates during recovery from a single bout of resistance-type exercise. We compared the effect of ingesting 20 g low glycated milk protein plus 2 g free leucine, or 20 g high glycated milk protein plus 2 g free leucine, or a noncaloric placebo, on the postprandial muscle protein synthetic response. By using intravenous infusion of L-[*ring*-^13^C_6_]-phenylalanine and collecting blood and muscle samples, we were able to evaluate postprandial whole-body and skeletal muscle protein synthesis rates.

## Methods

### Subjects

A total of 45 healthy, young males were recruited to participate in this double-blinded, randomized controlled study with 3 parallel treatments. Potential subjects were included if they were nonsmoking, recreationally active (exercise ≥1 h and ≤6 h · wk^-1^, with a maximum of 2 h · wk^-1^ resistance-type exercise), and did not regularly consume protein supplements (e.g., protein powders). The trial was conducted between September 2020 and January 2022 at Maastricht University Medical Centre+ in Maastricht, the Netherlands. Subjects were fully informed about the experimental procedures and possible risks of participation before signing an informed consent. The study was approved by the Medical Ethical Committee of Maastricht University Medical Centre, the Netherlands, and was registered at the Dutch Trial Register (NL8690). All procedures were carried out in accordance with the standards stated in the most recent version of the Helsinki Declaration. The study was independently monitored by the Clinical Trial Center Maastricht.

### Study design

This double-blind, randomized parallel study assessed the postprandial muscle protein synthetic response following the ingestion of 20 g low or high glycated milk protein or a noncaloric placebo after a single bout of resistance-type exercise. Subjects were randomly assigned to ingest 20 g of low glycated milk protein plus 2 g free leucine (LOW-GLYC), or 20 g of high glycated milk protein plus 2 g free leucine (HIGH-GLYC), or a noncaloric placebo (PLA). Free leucine (2 g) was added to the protein treatments to provide a potent anabolic signal for muscle protein synthesis [11]. Randomization for treatments was balanced, and a random allocation sequence was generated using www.randomization.com. An independent person was responsible for blinding and drink preparation. The randomization was not shared with investigators, study staff, or participants until all procedures and statistical analyses were completed. During experimental days, subjects ingested the investigational drink, followed by a 6 h postprandial assessment period during which multiple blood samples and muscle biopsies were collected.

### Pretesting

During screening, body weight and height were assessed, and body composition was determined by a dual-energy X-ray absorptiometry scan (DEXA; Discovery A; Hologic). All subjects were deemed healthy based on their responses to a routine medical screening questionnaire. Subsequently, subjects were familiarized with the resistance-type exercise protocol and the exercise equipment. All exercises during pretesting and experimental trials were supervised by trained personnel. Subjects started by performing a 5 min cycling warm-up at 100 W before completing an estimation of their 1-repetition maximum (1RM) on the leg press, leg extension, lateral pulldown, and chest press using the multiple-repetitions testing procedure [31]. For each exercise, subjects performed 10 submaximal repetitions to become familiarized with the equipment and to have lifting technique critiqued and properly adjusted. Sets were then performed at progressively increasing loads until failure to perform a valid 1RM estimation within 3–6 sets. A repetition was valid if the subject was able to complete the entire lift in a controlled manner without assistance. A 2-min resting period between subsequent attempts was allowed. The pretesting and experimental trials were separated by ≥7 days.

### Standardization of physical activity and diet

Subjects refrained from strenuous physical activity in the 48 h leading up to the experimental day. Physical activity and dietary intake were recorded 48 h prior to the experimental day. Subjects did not consume alcohol for 24 h and caffeine for 12 h before the experimental day. On the evening prior to the experimental day, all subjects consumed a standardized dinner (54 kJ/kg), providing 60% energy as carbohydrate, 25% energy as fat, and 15% energy as protein. After 22:00, subjects were only allowed to drink water.

### Milk protein intervention

**FIGURE 1:**
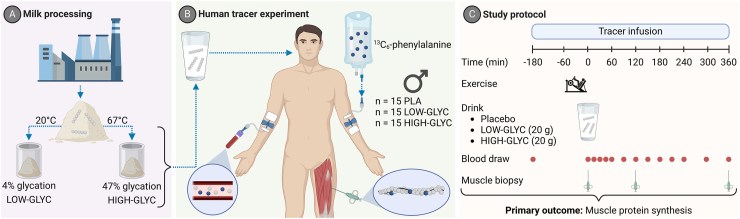
Experimental protocol. Milk was processed to skimmed milk powder and temporarily stored at different temperatures to obtain the desired glycation level (A). In turn, 45 adult, male subjects underwent an intravenous infusion with L-[*ring*-^13^C_6_]-phenylalanine tracer, followed by a single bout of whole-body resistance-type exercise, whereafter they ingested either 20 g of low glycated milk protein plus 2 g free leucine, 20 g of high glycated milk protein plus 2 g free leucine, or a noncaloric placebo (B). Frequent blood sample collection in combination with skeletal muscle biopsies during a 6 h postprandial period allowed the assessment of plasma amino acid concentrations and mixed muscle protein synthesis rates (C). This figure is created with BioRender.com. HIGH-GLYC, milk protein with a high glycation level; LOW-GLYC, milk protein with a low glycation level; PLA, placebo.

The milk protein powder consisted of a skimmed milk powder with a protein content of 37% (%N × 6.38) and a whey:casein protein ratio of 20:80, a lactose content of 47%, a fat content of 1%, a mineral content of 8%, and a moisture content of 4% (FrieslandCampina Innovation Centre). A single batch of skimmed milk powder was received from the factory and canned under stable conditions. To produce a high glycated milk powder, cans containing the skimmed milk powder were incubated in a stove (67°C, 336 h; Figure 1A). Lysine blockage was assessed by acid hydrolysis of the milk powders followed by quantification of lysine and furosine content using ion-pair reversed-phase HPLC (Qlip B.V. - Ansynth) [32]. Furosine is formed during acid hydrolysis of protein-bound Amadori products [33]. The fact that furosine does not occur naturally in dairy products makes it a reliable marker for lysine blockage when assessed under controlled conditions [23]. Table 1 describes the furosine content, blocked lysine level, lysine loss, and amino acid composition of the milk powders. On the experimental days, 54 g of milk powder and 2 g of crystalline leucine (L-Leucine, Amino GmbH) were dissolved in 300 mL water to provide a total of 20 g protein. 41 mg free L-[*ring*-^13^C_6_]-phenylalanine (4.5% of total phenylalanine in the drink, Cambridge Isotope Laboratories) was added to the milk beverages to prevent precursor pool dilution [34]. The noncaloric PLA consisted of 300 mL of water. All beverages were flavored with 1.5 mL vanilla flavor (Dr. Oetker) and provided in a nontransparent shaker bottle.

**TABLE 1:** Furosine content, lysine content, calculated blocked lysine and total lysine loss and amino acid composition for the LOW-GLYC and HIGH-GLYC milk protein powders.

	LOW-GLYC	HIGH-GLYC
Furosine (mmol/kg powder)	3	28
Total lysine (mmol/kg powder)	217	202
Lysine as reactive lysine (mmol/kg powder)	209	116
Lysine as Amadori product (mmol/kg powder)	8	86
Lysine loss due to advanced Maillard (mmol/kg powder)	—	15
Blocked lysine (%)	4	43
Total lysine loss (%)	4	47

AA (% of total)	LOW-GLYC & HIGH-GLYC	

Alanine	3.0	
Arginine	3.3	
Aspartic acid	7.4	
Cystine	0.7	
Glutamic acid	20.9	
Glycine	1.8	
Histidine	2.5	
Isoleucine	5.0	
Leucine (without additional 2 g leucine supplementation)	9.4	
Lysine (before heat-induced glycation)	8.8	
Methionine	2.5	
Phenylalanine	4.7	
Proline	8.5	
Serine	5.2	
Threonine	4.1	
Tryptophan	1.4	
Tyrosine	4.7	
Valine	6.1	

EAA	44.3	
NEAA	55.7	

TAA	100.0	

Blocked lysine is calculated as the percentage of total lysine in the specific powder. Total lysine loss is calculated as the percentage of total lysine in the untreated powder (i.e., LOW-GLYC). Abbreviations: HIGH-GLYC, milk protein with a high glycation level; LOW-GLYC, milk protein with a low glycation level.

### Tracer preparation

The stable isotope tracers L-[*ring*-^13^C_6_]-phenylalanine and L-[*ring*-3,5-^2^H_2_]-tyrosine were purchased from Cambridge Isotope Laboratories and dissolved in 0.9% saline before infusion (A15 Pharmacy). Continuous intravenous infusion was performed using a calibrated IVAC 598 pump.

### Experimental procedures

An outline of the study protocol is provided in Figure 1B–C. At 08:00, subjects reported to the laboratory after an overnight fast. The day started with the placement of a polytetrafluoroethylene catheter into an antecubital vein for stable-isotope amino acid infusion and a second polytetrafluoroethylene catheter in a dorsal hand vein of the contralateral arm for arterialized blood draws. The hand was first placed in a hot box (60°C) for 10 min before drawing blood to allow sampling of arterialized blood (10 mL blood per sample collected). After collection of a basal blood sample (*t* = −180 min, time expressed relative to intake of protein drink at *t* = 0 min), the plasma phenylalanine and tyrosine pools were primed with a single intravenous dose of L-[*ring*-^13^C_6_]-phenylalanine (2.7 μmol/kg) and L-[*ring*-3,5-^2^H_2_]-tyrosine (0.9 μmol/kg). Once primed, the continuous stable isotope infusion of L-[*ring*-^13^C_6_]-phenylalanine (0.06 μmol/kg/min) and L-[*ring*-3,5-^2^H_2_]-tyrosine (0.02 μmol/kg/min) was initiated and lasted until the end of the experimental day. Following the start of infusion, subjects rested in a supine position for 120 min, where after a single bout of whole-body resistance-type exercise was performed (*t* = −60 min). Directly following exercise, an arterialized blood sample and the first muscle biopsy were collected (*t* = 0 min). Subjects then consumed the given test drink within 5 min, followed by a 6 h postprandial period. During the postprandial period, blood samples were collected at *t =* 15, 30, 45, 60, 90, 120, 150, 180, 210, 240, 300, and 360 min while subjects rested in a supine position. At *t* = 120 min, a second muscle biopsy was obtained proximal to the first biopsy. A third biopsy was obtained from the contralateral leg at *t =* 360 min. Subjects were allowed to drink water *ad libitum*.

### Exercise protocol

The exercise protocol consisted of ∼60 min of whole-body resistance-type exercise. After a 5 min cycling warm-up at 100 W, subjects performed 4 sets on the horizontal leg press, leg extension, lateral pulldown, and chest press. For all exercises, the first set consisted of 10 repetitions at 50% of the subjects’ 1RM to warm up on the equipment. The second and third sets were performed at 80% 1RM, aiming at 7–9 repetitions. The last set was performed at 80% 1RM until volitional exhaustion [35]. Load was adjusted if less than 6 or more than 10 repetitions could be completed. Two-minute rest intervals were used between all sets.

### Plasma and muscle analysis

Blood samples were collected in tubes containing EDTA and centrifuged at 1000 × *g* for 10 min at 4°C. Aliquots of plasma were frozen in liquid nitrogen and stored at −80°C. Muscle biopsies were obtained from the middle region of the *vastus lateralis* muscle, 15 cm above the patella and ∼4 cm below entry through the fascia, using the percutaneous needle biopsy technique [36]. Muscle samples were dissected carefully and freed from any visible nonmuscle material. The muscle samples were immediately frozen in liquid nitrogen and stored at −80°C until further analysis.

Plasma glucose and insulin concentrations were analyzed using commercially available kits (ABX Pentra Glucose HK CP, Horiba ABX, ref: A11A01667 and Human Insulin kit, Meso Scale Discovery, ref: K151BZC). Plasma amino acid concentrations and plasma L-[*ring*-^13^C_6_]-phenylalanine, L*-*[*ring-*^13^C_6_]-tyrosine, and L-[*ring*-3,5-^2^H_2_]-tyrosine enrichments were determined by UPLC-MS. Plasma amino acids were derivatized to its 6-aminoquinolyl-N-hydroxysuccinimidyl carbamate derivative, and concentrations were determined, as described previously [17]. Phenylalanine and tyrosine enrichments were determined by UPLC-MS by using mass detection of masses 336, 342, and 346 for unlabeled and (^13^C_6_ and ^13^C_9_-^15^N)-labeled phenylalanine, respectively, and 466, 468, 472, and 476 for unlabeled and (^2^H_2_, ^13^C_6_, and ^13^C_9_-^15^N)-labeled tyrosine, respectively. We applied standard calibration curves in all isotopic enrichment analyses to assess the linearity of the mass spectrometer and to control for the loss of tracer.

Enrichment of L-[*ring*-^13^C_6_]-phenylalanine in mixed muscle protein was determined by GC-C-IRMS (MAT 253; Thermo Fisher), as described previously [37].

### Calculations

Protein glycation level was assessed by quantification of lysine and furosine content following acid hydrolysis of the milk powders. Total lysine content of the milk powder, blocked lysine level, and the total lysine lost were calculated as follows:

$$Total\;lysine\;\left(reactive+blocked\right)={lysine}_{acid}+1.87\cdot furosine$$

$$Blocked\;lysine\;\left(\%\right)=\frac{3.1\cdot furosine}{total\;lysine}\cdot 100$$

$$Total\;lysine\;loss\;\left(\%\right)=\frac{3.1\cdot {furosine}_{HIGH}+\left({total\;lysine}_{LOW}-{total\;lysine}_{HIGH}\right)}{{total\;lysine}_{LOW}}\cdot 100$$

Lysine_acid_ represents the lysine content measured following acid hydrolysis of the milk powder. Furosine_HIGH_ represents the furosine content measured in the HIGH-GLYC milk powder. Total lysine_LOW_ represents the total lysine content in the LOW-GLYC milk powder. Total lysine_HIGH_ represents the total lysine content in the HIGH-GLYC milk powder.

Whole-body protein metabolism was based on standard plasma phenylalanine kinetics calculations with the amount of exogenous phenylalanine appearing into the circulation estimated as recently described and calculations corrected for the small amount of oral free L-[*ring*-^13^C_6_]-phenylalanine that was coingested [16,38] (a full description of the calculations is provided in the Supplemental Methods).

Mixed muscle protein fractional synthetic rate (FSR) was calculated using the standard precursor-product equation as follows:

$$FSR=\frac{\Delta E_{p}}{E_{precursor}\cdot t}\cdot 100$$

where ΔE_p_ is the Δ increment of protein-bound L-[*ring*-^13^C_6_]-phenylalanine during incorporation periods; E_precursor_ is the mean plasma L-[*ring*-^13^C_6_]-phenylalanine enrichment during the time period for determination of amino acid incorporation; *t* indicates the time interval (h) between biopsies; and factor 100 is used to express the FSR in percent per hour (% · h^-1^).

### Statistical analysis

A sample size of 15 subjects per group, including 5% drop-out, was calculated with a power of 80% and an α of 0.05/3 (Bonferroni correction for 3 treatment arms) to detect a difference of 0.03 % · h^-1^ in FSR rate between groups [39]. Experimental data of all subjects were included in the final analysis. The primary outcome parameter was muscle protein synthesis rate over the full 6 h postprandial period. Secondary outcome parameters included plasma glucose, insulin, total plasma amino acid (TAA), EAA, lysine, phenylalanine, and leucine concentrations, plasma L-[*ring*-^13^C_6_]-phenylalanine enrichments, whole-body protein metabolism (i.e., protein synthesis, breakdown, oxidation and net balance over the full postprandial period), and muscle protein synthesis rates over 0–120 and 120–360 min following ingestion of the protein. Normal distribution of all parameters was verified by Shapiro-Wilk tests and visual inspection of Q-Q plots. Time-dependent variables (i.e., plasma glucose, insulin and amino acid concentrations, and plasma enrichments) were analyzed by 2-factor repeated-measures ANOVA with time as a within-subjects factor and treatment group as between-subjects factor. In case of significant interactions, separate one-way ANOVAs were performed for each time period to detect differences between treatments. Bonferroni post hoc test was applied to locate group differences. Time-independent variables (i.e., peak lysine concentration, incremental AUC for plasma amino acid concentrations over the *t* = 0–360 min period, whole-body protein metabolism, and integrative FSR values for 0–120, 120–360, and 0–360 min) were analyzed using a 2-tailed, one-way ANOVA with treatment as between-subject factor. In case of a significant main effect, Bonferroni post hoc test was applied to locate group differences. Statistical significance was set at *P* < 0.05. All calculations were performed using SPSS Statistics (version 27; IBM) and are presented as mean values with their standard deviations.

## Results

### Subject inclusion and characteristics

The participant flow chart is presented in Supplemental Figure 1. 53 participants were included in the study, of which 46 participated in an experimental day. One of these participants was randomized but did not receive the allocated intervention because he felt nauseous during the exercise protocol and could not continue the experiment. Thus, 45 participants completed the experiment. Data from all 45 participants who completed the experimental protocol were included in the final analyses. Subjects’ characteristics are presented in Table 2.

**Table 2:** Subjects’ characteristics.

	PLA (*n* = 15)	LOW-GLYC (*n* = 15)	HIGH-GLYC (*n* = 15)
Age (y)	24 ± 4	23 ± 4	24 ± 4
Weight (kg)	74.6 ± 10.0	70.5 ± 9.1	72.9 ± 12.8
Height (m)	1.78 ± 0.08	1.80 ± 0.05	1.77 ± 0.09
BMI (kg·m^-2^)	23.4 ± 2.0	21.6 ± 2.2	23.2 ± 2.4
LBM (kg)	55.4 ± 9.0	54.2 ± 6.1	54.7 ± 8.2
Body fat (%)	22 ± 5	20 ± 4	21 ± 4
1RM leg press (kg)	202 ± 50	208 ± 54	217 ± 43
1RM leg extension (kg)	69 ± 15	65 ± 10	69 ± 16
1RM lat pulldown (kg)	106 ± 23	109 ± 13	106 ± 24
1RM chest press (kg)	79 ± 26	64 ± 14	77 ± 23

All values are means ± SD. BMI, body mass index; HIGH-GLYC, milk protein with a high glycation level; LBM, lean body mass; LOW-GLYC, milk protein with a low glycation level; PLA, placebo; 1RM, one repetition max.

### Plasma glucose and insulin concentrations

**FIGURE 2:**
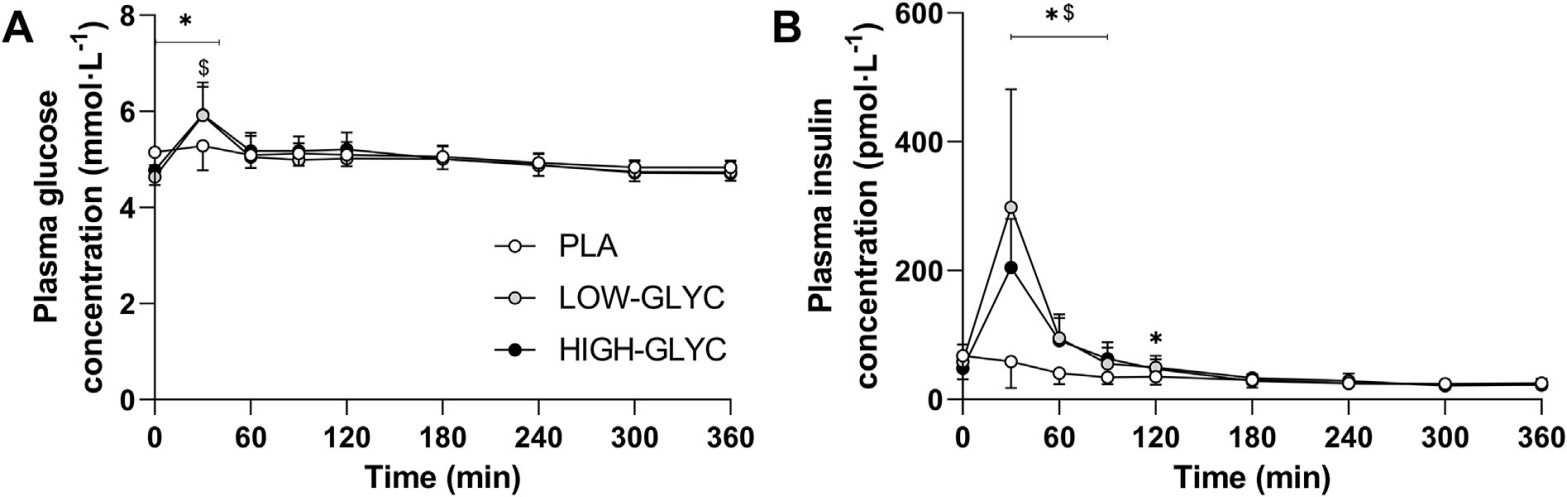
Plasma glucose (A) and insulin (B) concentrations following ingestion of LOW-GLYC, HIGH-GLYC, and PLA. Data are analyzed by 2-factor repeated measures ANOVA with time as within-subject factor and treatment as between-subject factor. In case of significant interactions, separate one-way ANOVAs were performed for each time period to detect differences between treatments. Bonferroni post hoc test was applied to locate group differences. ∗ indicates LOW-GLYC significantly different from PLA; $ indicates HIGH-GLYC significantly different from PLA. Data are expressed as mean ± standard deviation. HIGH-GLYC, milk protein with a high glycation level; LOW-GLYC, milk protein with a low glycation level; PLA, placebo.

Plasma glucose and insulin concentrations are depicted in Figure 2. Plasma glucose concentrations showed a small, transient increase following protein ingestion (time × treatment effect: *P* < 0.001). Plasma insulin concentrations increased following protein ingestion and were higher in the protein groups compared with PLA (time × treatment effect: *P* < 0.001). For both plasma glucose and insulin concentrations, no differences were observed between LOW-GLYC and HIGH-GLYC.

### Plasma amino acid concentrations

**FIGURE 3:**
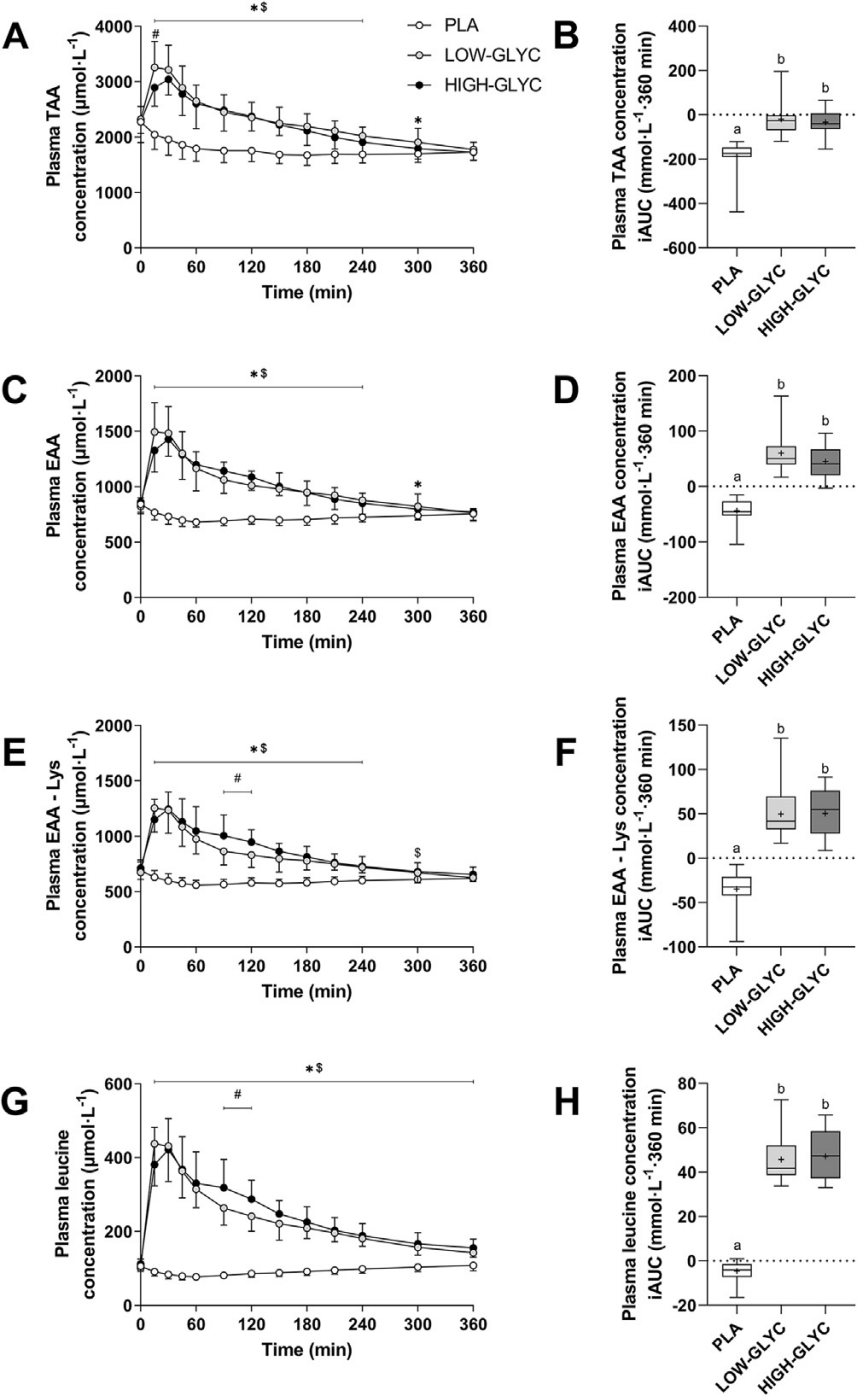
Plasma total amino acid concentrations (TAA; A), essential amino acid concentrations (EAA; C), essential amino acid concentrations without lysine (EAA – Lys; E), leucine concentrations (G) and their respective incremental areas under the curves (iAUCs; B, D, F, and H) following ingestion of LOW-GLYC, HIGH-GLYC, and PLA. Plasma concentrations are analyzed by 2-factor repeated measures ANOVA with time as within-subject factor and treatment as between-subject factor. In case of significant interactions, separate one-way ANOVAs were performed for each time period to detect differences between treatments. Bonferroni post hoc test was applied to locate group differences. iAUCs are analyzed by one-way ANOVA with Bonferroni post hoc test to locate differences between treatments. Plasma concentration data: ∗ indicates LOW-GLYC significantly different from PLA; $ indicates HIGH-GLYC significantly different from PLA; # indicates LOW-GLYC significantly different from HIGH-GLYC. iAUC data: treatments without a common letter differ, *P* < 0.05. Data in Figures 3A, 3C, 3E, and 3G are expressed as mean ± SD. Data in Figures 3B, 3D, 3F, and 3H are expressed as box and whisker plots with the median (line), mean (cross), interquartile range (box), and minimum and maximum values (tails). HIGH-GLYC, milk protein with a high glycation level; LOW-GLYC, milk protein with a low glycation level; PLA, placebo.

Protein ingestion resulted in an increase in TAA concentrations up to 120 min following ingestion of the protein. Higher plasma TAA concentrations were observed following LOW-GLYC and HIGH-GLYC ingestion compared with PLA (time × treatment effect: *P* < 0.001, Figure 3A). In line, incremental AUCs (iAUCs) for plasma TAA concentrations over the full 6 h postprandial period were higher for LOW-GLYC and HIGH-GLYC compared with PLA but did not differ between the protein treatments (−187 ± 74 vs −17 ± 77 vs −34 ± 60 mmol · L^-1^ · 360 min, for PLA, LOW-GLYC and HIGH-GLYC, respectively, treatment effect: *P* < 0.001, Figure 3B).

Plasma concentrations of EAA and EAAs without lysine (EAA – Lys) and their iAUCs are shown in Figure 3C–F. Plasma EAA concentrations and its iAUC over the full 6 h postprandial period were higher for LOW-GLYC and HIGH-GLYC compared with PLA (time × treatment effect: *P* < 0.001, iAUC treatment effect: *P* < 0.001), but no differences were observed between LOW-GLYC and HIGH-GLYC. In contrast, when lysine was excluded from the EAAs, plasma EAA-Lys concentrations for HIGH-GLYC were slightly higher compared with LOW-GLYC between 90 and 120 min (*P* ≤ 0.018 for both timepoints, Figure 3E), but this did not result in a different iAUC between the protein treatments over the full postprandial period (*P* = 1.00, Figure 3F). In addition, plasma leucine concentrations were higher for LOW-GLYC and HIGH-GLYC compared with PLA (time × treatment effect: *P* < 0.001, Figure 3G). Plasma leucine concentrations were slightly higher for HIGH-GLYC compared with LOW-GLYC between 90 and 120 min, but the iAUC over the full 6 h period did not differ (*P* = 1.00, Figure 3H).

**FIGURE 4:**
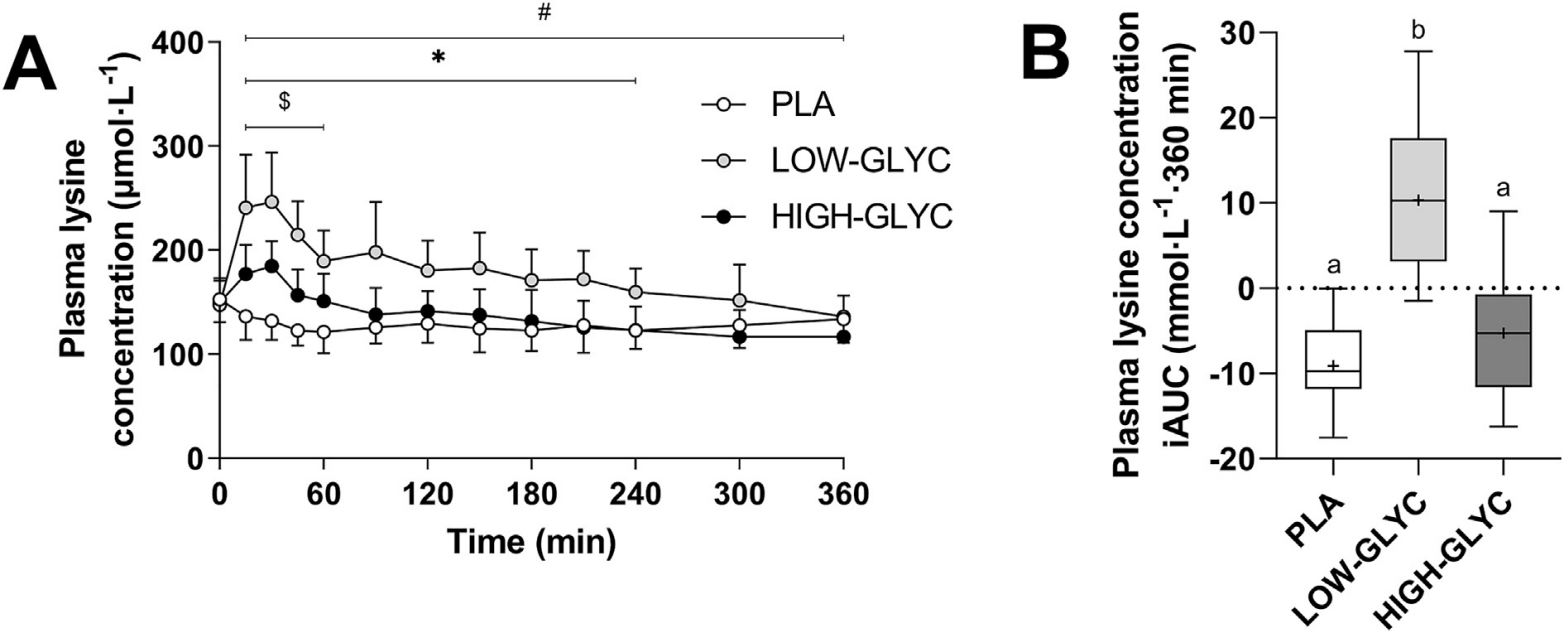
Plasma lysine concentrations (A) and the incremental area under the curve (iAUC; B) following ingestion of LOW-GLYC, HIGH-GLYC, and PLA. Plasma lysine concentrations are analyzed by 2-factor repeated measures ANOVA with time as within-subject factor and treatment as between-subject factor. In case of significant interactions, separate one-way ANOVAs were performed for each time period to detect differences between treatments. Bonferroni post hoc test was applied to locate group differences. iAUCs are analyzed by one-way ANOVA with Bonferroni post host test to locate differences between treatments. Plasma concentration data: ∗ indicates LOW-GLYC significantly different from PLA; $ indicates HIGH-GLYC significantly different from PLA; # indicates LOW-GLYC significantly different from HIGH-GLYC. iAUC data: treatments without a common letter differ, *P* < 0.05. Data in Figure 4A are expressed as mean ± SD. Data in Figure 4B are expressed as box and whisker plots with the median (line), mean (cross), interquartile range (box), and minimum and maximum values (tails). HIGH-GLYC, milk protein with a high glycation level; LOW-GLYC, milk protein with a low glycation level; PLA, placebo.

Plasma lysine concentrations and its iAUC are depicted in Figure 4. Plasma lysine concentrations were higher for LOW-GLYC compared with HIGH-GLYC and PLA throughout almost the entire postprandial period (time × treatment effect: *P* < 0.001, Figure 4A). As a result, peak lysine concentrations were 28% lower following ingestion of HIGH-GLYC compared with LOW-GLYC (191 ± 23 vs 266 ± 47 μmol · L^-1^, respectively, *P* < 0.001). Still, ingestion of HIGH-GLYC resulted in a higher lysine concentration compared with PLA during the first 60 min after ingestion of the drink. Nevertheless, the iAUC of plasma lysine over the full 6 h postprandial period was only higher following ingestion of LOW-GLYC compared with HIGH-GLYC and PLA (−9 ± 5 vs 10 ± 9 vs −5 ± 7 mmol · L^-1^ · 360 min, for PLA, LOW-GLYC and HIGH-GLYC, respectively, treatment effect: *P* < 0.001, Figure 4B).

**FIGURE 5:**
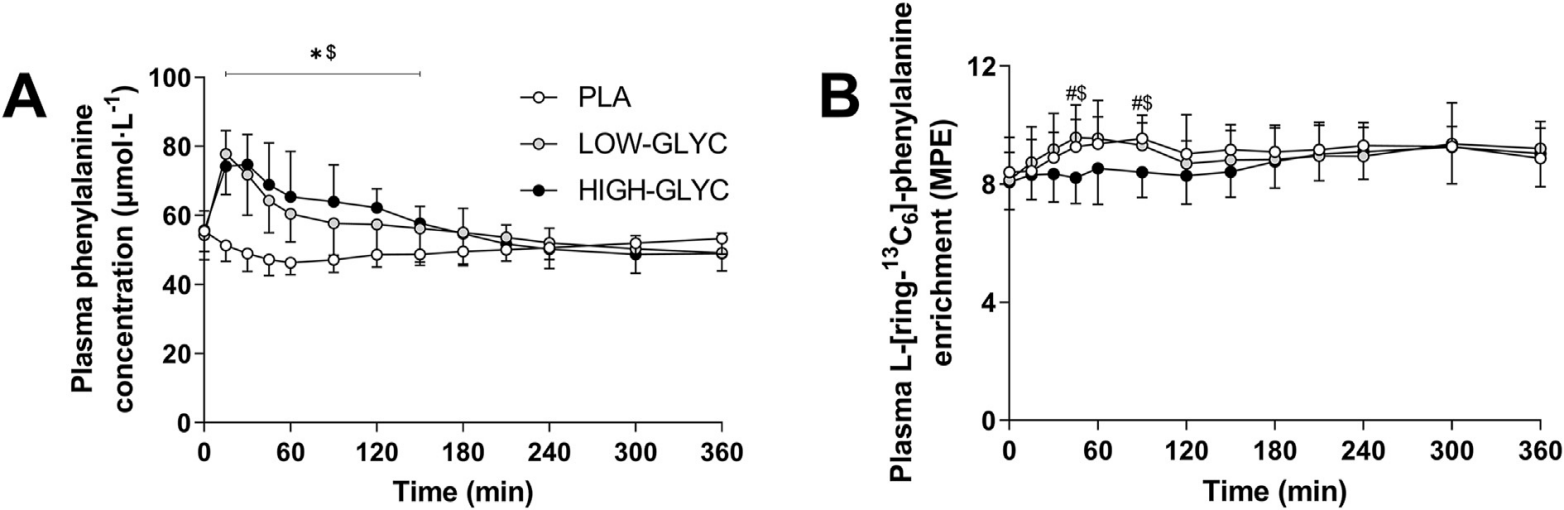
Plasma phenylalanine concentrations (A) and L-[*ring*-^13^C_6_]-phenylalanine enrichments (B) following ingestion of LOW-GLYC, HIGH-GLYC, and PLA. Plasma phenylalanine concentrations and L-[*ring*-^13^C_6_]-phenylalanine enrichments are analyzed by 2-factor repeated measures ANOVA with time as within-subject factor and treatment as between-subject factor. In case of significant interactions, separate one-way ANOVAs were performed for each time period to detect differences between treatments. Bonferroni post hoc test was applied to locate group differences. ∗indicates LOW-GLYC significantly different from PLA; $ indicates HIGH-GLYC significantly different from PLA; # indicates LOW-GLYC significantly different from HIGH-GLYC. Data are expressed as mean ± SD. HIGH-GLYC, milk protein with a high glycation level; LOW-GLYC, milk protein with a low glycation level; MPE, mole percent excess; PLA, placebo.

Figure 5A shows the postprandial plasma phenylalanine concentrations following ingestion of the three beverages. LOW-GLYC and HIGH-GLYC resulted in higher plasma phenylalanine concentrations until *t* = 150 min compared with PLA (time × treatment effect: *P* < 0.001). An overview of the postprandial responses of non-essential amino acids, branched-chain amino acids, and all other individual amino acids is provided in Supplemental Figure 2.

### Plasma L-[ring-^13^C_6_]-phenylalanine enrichments

Plasma L-[ring-^13^C_6_]-phenylalanine enrichments did not differ between treatments before ingestion of the drinks (*t* = 0 min, *P* = 0.628). Plasma L-[ring-^13^C_6_]-phenylalanine enrichments slightly increased following ingestion of LOW-GLYC and PLA and were higher than HIGH-GLYC at *t* = 45 and *t* = 90 min (time × treatment effect: *P* < 0.001, Figure 5B).

### Whole-body protein metabolism

**FIGURE 6:**
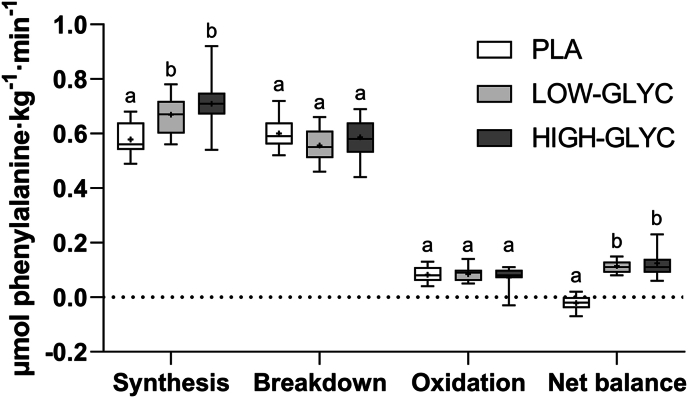
Whole-body protein synthesis, breakdown, oxidation, and net protein balance over the entire 6 h postprandial period following ingestion of LOW-GLYC, HIGH-GLYC, or PLA (*n* = 15 for each group). Data are analyzed by a one-way ANOVA with treatment as between-subject factor. Treatments without a common letter differ, *P* < 0.05. The data are presented as box and whisker plots with the median (line), mean (cross), interquartile range (box), and minimum and maximum values (tails). HIGH-GLYC, milk protein with a high glycation level; LOW-GLYC, milk protein with a low glycation level; PLA, placebo.

Whole-body protein metabolism based on phenylalanine kinetics is presented in Figure 6. Whole-body protein synthesis was higher following the ingestion of LOW-GLYC and HIGH-GLYC compared to PLA but did not differ between the protein treatments. No differences in whole-body protein breakdown and amino acid oxidation were observed between treatments. As a result, whole-body net balance was higher following ingestion of LOW-GLYC and HIGH-GLYC compared with PLA, with no difference between the protein treatments (−0.02 ± 0.03 vs 0.11 ± 0.02 vs 0.12 ± 0.05 μmol phenylalanine · kg^-1^ · min^-1^ for PLA, LOW-GLYC, and HIGH-GLYC, respectively, treatment effect: *P* < 0.001, treatment effects after Bonferroni: *P* < 0.001).

### Muscle protein synthesis rates

**FIGURE 7:**
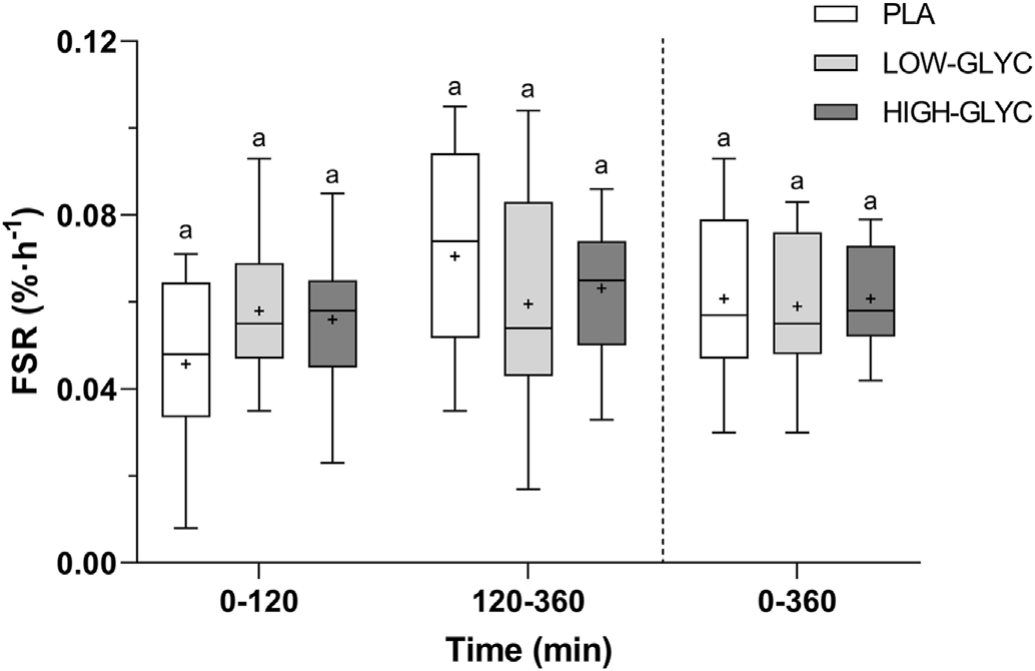
Mixed muscle protein synthesis rates following ingestion of LOW-GLYC, HIGH-GLYC, and PLA. Data are analyzed by a one-way ANOVA with treatment as between-subject factor. Treatments without a common letter differ, *P* < 0.05. The data are presented as box and whisker plots with the median (line), mean (cross), interquartile range (box), and minimum and maximum values (tails). FSR, fractional synthetic rate; HIGH-GLYC, milk protein with a high glycation level; LOW-GLYC, milk protein with a low glycation level; PLA, placebo.

Postprandial muscle protein synthesis rates are shown in Figure 7. Muscle protein synthesis rates did not differ between treatments over the full 6 h postprandial period (0.061 ± 0.018 vs 0.059 ± 0.016 vs 0.061 ± 0.012 % · h^-1^ for PLA, LOW-GLYC, and HIGH-GLYC, respectively, treatment effect: *P* = 0.939). In line, muscle protein synthesis rates were not different between treatments during the first 2 h (0.046 ± 0.020 vs 0.058 ± 0.016 vs 0.056 ± 0.016 % · h^-1^ for PLA, LOW-GLYC, and HIGH-GLYC, respectively, treatment effect: *P* = 0.138) and between 2 and 6 h following intake of the drinks (0.071 ± 0.024 vs 0.060 ± 0.024 vs 0.063 ± 0.016 % · h^-1^ for PLA, LOW-GLYC, and HIGH-GLYC, respectively, treatment effect: *P* = 0.385).

## Discussion

The present study assessed the impact of dietary protein glycation on postprandial plasma amino acid availability and muscle protein synthesis rates during recovery from a single bout of whole-body resistance-type exercise. Ingestion of high glycated milk protein resulted in a lower plasma lysine availability compared with the ingestion of low glycated milk protein. However, the muscle protein synthetic response did not differ following the ingestion of 20 g low or high glycated milk protein or a noncaloric placebo.

Previous studies using *in vitro* and animal models have demonstrated that protein glycation impairs protein digestion [24,[40], [41], [42], [43]]. *In vitro*, gastrointestinal models suggest that protein glycation decreases the ability of digestive enzymes to hydrolyze the protein and effectively liberate lysine [24,40,44]. In support, we have shown that protein glycation attenuates postprandial plasma lysine concentrations *in vivo* in humans in a dose-dependent manner [17]. Moreover, we have recently applied specifically produced, intrinsically labeled protein to prove that the attenuated rise in postprandial plasma lysine concentrations following ingestion of protein with a high versus low glycation level is attributed to a lower postprandial bioavailability of milk protein-derived lysine *in vivo* in humans [26]. In agreement with our previous work, our current data demonstrate an attenuated postprandial rise in circulating plasma lysine concentrations following the ingestion of high versus low glycated milk protein during postexercise recovery (Figure 4). Over the full postprandial period, the ingestion of low glycated milk protein resulted in a higher plasma lysine availability, whereas the ingestion of high glycated milk protein did not elevate plasma lysine availability compared with placebo. Altogether, these data underline that simply the heating of milk protein, comparable with the level of processing that is observed in commercial products [27,30], can substantially compromise postprandial plasma lysine (bio)availability.

Protein quality is often defined as the protein’s capacity to provide all the EAAs in sufficient bioavailable amounts to meet their requirements. In addition to the effect on lysine, we examined whether protein glycation affects the postprandial availability of other amino acids. Protein glycation did not attenuate the availability of the sum of all other EAAs or, more specifically, leucine and phenylalanine. In fact, the sum of all EAA concentrations (minus lysine) and leucine concentrations were even slightly elevated for a short period following the ingestion of high versus low glycated protein (Figure 3). We recently observed a similar effect of protein glycation on protein-derived phenylalanine and leucine release into the circulation [26]. A potential explanation may be that the lower lysine availability as a result of protein glycation results in an unbalanced amino acid supply (i.e., specific lysine deficiency) to the splanchnic bed, thereby lowering splanchnic protein synthesis rates, resulting in more of the absorbed amino acids being released into the circulation [45,46]. Altogether, our data demonstrate that protein glycation specifically attenuates the bioavailability of the amino acid lysine. A low postprandial plasma lysine availability is typical for many plant-based protein sources, which are generally considered to be of low(er) quality [47]. Milk protein is considered a high-quality protein based on its high EAA content, well-balanced amino acid profile, and high digestibility. Protein glycation may substantially lower the *in situ* quality of milk protein to a level that no longer differs from several plant-derived proteins that are deficient in lysine. As such, processing can have enormous impact on the lysine bioavailability of a protein or protein source and should, therefore, be considered when protein quality is evaluated.

In the present study, we extend on our previous work on bioavailability by also assessing the impact of protein glycation level on the biofunctionality of the protein. Ingestion of both the low and high level glycated protein increased postprandial amino acid availability, which resulted in an increase in whole-body protein synthesis rates compared with the placebo treatment (Figure 6). This allowed postprandial whole-body net protein balance to become positive during the postexercise recovery period. Despite the large difference in postprandial plasma lysine availability, we observed no differences in whole-body net protein balance following the ingestion of high versus low glycated protein. The lower plasma lysine availability resulting from protein glycation does not seem to restrict the whole-body protein synthetic response to protein ingestion. As whole-body protein metabolism does not necessarily reflect skeletal muscle metabolism, we also collected muscle biopsies before and after protein ingestion to assess the effect of protein glycation level on the postexercise muscle protein synthetic response to protein ingestion.

Adequate availability of all EAAs is required for a sustained postprandial muscle protein synthetic response [14]. In our previous work, we observed a 63% reduction in protein-derived lysine availability as a result of ∼50% protein glycation [26]. Based on this work, the low and high glycated milk protein in the present study would have a Digestible Indispensable Amino Acid Score (DIAAS) of >100 and 70, respectively. Therefore, we hypothesized that the lower availability of lysine following ingestion of high glycated milk protein would result in a lower muscle protein synthetic response compared with low glycated milk protein. In contrast to our hypothesis, we observed no differences between low and high glycated protein ingestion on postexercise muscle protein synthesis rates (Figure 7). These data imply that impaired postprandial dietary protein-derived lysine availability does not restrict the capacity to increase postexercise muscle protein synthesis rates. These results seem to agree with other studies that failed to report lower postprandial muscle protein synthesis rates following the ingestion of plant-derived proteins that were low in lysine, when compared with high-quality animal-derived protein sources in healthy young adults [[48], [49], [50], [51]]. We speculate that the endogenous release of lysine may provide sufficient lysine as a precursor to support the postprandial increase in muscle protein synthesis both at rest as well as during recovery from exercise. In contrast to prior work by many [8,9,52,53], but certainly not all [[3], [4], [5]], we failed to detect an effect of ingesting either low or high glycated protein on postexercise muscle protein synthesis rates compared with the placebo treatment. Apparently, the 20 g protein dose did not suffice to allow a detectable increase in muscle protein synthesis. We can only speculate whether provision of a larger protein dose could have resulted in a difference in the anabolic response following ingestion of low or high concentration glycated protein.

Only recently, interest has been directed toward food processing and its impact on protein bioavailability and biofunctionality. The present study is one of the first to investigate the impact of food processing (i.e., protein glycation) on protein biofunctionality *in vivo* in humans. Although we did not observe an effect of protein glycation on the acute anabolic properties of the protein, the compromised protein quality may become a health issue under conditions where the diet is characterized by low protein and/or low lysine intake, chronic consumption of glycated protein products, and in populations with elevated lysine requirements [[54], [55], [56], [57], [58], [59], [60]]. Moreover, the supply of dietary lysine has been suggested to become a critical factor in the global protein transition [61,62]. Therefore, more focus needs to be placed on the impact of food processing and protein extraction procedures on the actual nutritional quality of a protein source by assessing protein bioavailability and biofunctionality of our processed foods and food ingredients in an *in vivo* human setting.

In conclusion, ingestion of dietary protein with a high glycation level lowers postprandial plasma lysine availability *in vivo* in humans. The lower lysine availability as a result of protein glycation does not seem to modulate the acute postprandial muscle protein synthetic response during recovery from exercise in healthy, young males. Industrial processing and storage of (milk) products can modulate the bioavailability of a protein source and, as such, compromise *in situ* protein quality.

## Author contributions

The authors’ responsibilities were as follows – GAAvL, JT, JN, MCEB, LJCvL: designed research; GAAvL, JT, FKH, JMS: conducted research; JvK, JMS, JPBG: performed sample analyses; GAAvL, JT, LBV: performed statistical analysis; GAAvL, JT, LJCvL: wrote the manuscript and had primary responsibility for final content; and all authors: read and approved the final manuscript.

## Data availability

Data described in the manuscript, code book, and analytic code will be made available upon request, pending application and approval.

## Funding

The present study was supported (in part) by FrieslandCampina, the Netherlands, and TKI Agri & Food, the Netherlands. LJCvL reports financial support was provided by TKI Agri & Food. LJCvL reports financial support was provided by FrieslandCampina. The supporting sources had no involvement in data collection and analyses nor imposed restrictions regarding publication of the work.

## Conflict of interest

GAAvL reports a relationship with FrieslandCampina that includes employment. JN reports a relationship with FrieslandCampina that includes employment. MCEB reports a relationship with FrieslandCampina that includes employment. JT reports a relationship with FrieslandCampina that includes consulting or advisory, funding grants, and speaking and lecture fees. LBV reports a relationship with FrieslandCampina that includes consulting or advisory, funding grants, and speaking and lecture fees. LJCvL reports a relationship with FrieslandCampina that includes consulting or advisory, funding grants, and speaking and lecture fees. All other authors report no conflicts of interest.
