# Supplementary material for: Milk Protein Glycation Compromises Postprandial Lysine Bioavailability but does not Modulate Postprandial Muscle Protein Synthesis Rates In Vivo in Males: A Double-blind, Randomized Parallel Trial
Source: J Nutr. 2025 May 27;155(7):2215–26. doi: 10.1016/j.tjnut.2025.05.032 (PMC12308134; doi:10.1016/j.tjnut.2025.05.032)

**Milk protein glycation strongly compromises post-prandial lysine bioavailability, but does not modulate post-prandial muscle protein synthesis rates *in vivo* in males: a double-blind randomized parallel trial**

Glenn AA van Lieshout^1,2^, Jorn Trommelen^1^, Floris K Hendriks^1^, Jean Nyakayiru^2^, Janneau van Kranenburg^1^, Joan M Senden^1^, Joy PB Goessens^1^, Lex B Verdijk^1^, Marjolijn CE Bragt^2^, Luc JC van Loon^1,*^

*^1^Department of Human Biology, NUTRIM Institute of Nutrition and Translational Research in Metabolism, Maastricht University Medical Centre+, Maastricht, the Netherlands*

*^2^FrieslandCampina, Amersfoort, the Netherlands*

SUPPLEMENTARY DATA

**SUPPLEMENTAL METHODS.**

The exogenous appearance over the full 6 h postprandial period was estimated for the ingested milk protein (EXO_pro_) and the ingested free L-[*ring*-^13^C_6_]-phenylalanine (EXO_oral tracer_) as follows.

$$EXO=\frac{{EXO}_{(\%)}}{BM\cdot100}\cdot{AA}_{oral}$$

EXO, represent the amount of exogenous phenylalanine or L-[*ring*-^13^C_6_]-phenylalanine that becomes available in the circulation over the 6 h post-prandial period expressed in µmol·kg BM^-1^·360 min^-1^. EXO_(%)_ represents the percentage of (dietary protein-derived) amino acids appearing into the circulation over 6 h, which was estimated at 58% for the ingested milk protein (37, 50), and 76% for the ingested free L-[*ring*-^13^C_6_]-phenylalanine (37, 60). BM represents the body mass of the participant. 100 is used to convert EXO_(%)_ from a percentage to a fraction. AA_oral_ represents the ingested amount of amino acid (i.e., phenylalanine or free L-[*ring*-^13^C_6_]-phenylalanine) in µmol.

Intravenous infusion of L-[*ring*-^13^C_6_]-phenylalanine combined with arterialized blood sampling allowed us to quantify various aspects of post-prandial protein handling (32). Formulas were modified to correct for the ingestion of a small amount of free L-[*ring*-^13^C_6_]-phenylalanine. The total and endogenous phenylalanine appearance (µmol·kg BM^-1^·360 min^-1^) and the total phenylalanine disappearance (µmol·kg BM^-1^·360 min^-1^) over the 6 h post-prandial period were calculated as follows.

$$Total appearance=\frac{F_{iv}+{EXO}_{oral tracer}-\left[ pV\cdot C\cdot\Delta E \right]}{E}$$

$$Endo appearance=protein breakdown =total appearance-{EXO}_{pro}-{EXO}_{oral tracer}{-F}_{iv}$$

$$Total disappearance=total appearance-pV\cdot\Delta C$$

F_iv_ represents the cumulative intravenous tracer infusion of L-[*ring*-^13^C_6_]-phenylalanine over the entire 6 h post-prandial period. EXO_oral tracer_ represents the cumulative exogenous plasma L-[*ring*-^13^C_6_]-phenylalanine bioavailability over the 6 h post-prandial period. pV (0.125 L·kg^-1^) represents the distribution volume (61). C represents the mean plasma phenylalanine concentration over the 6 h post-prandial period. ΔE represents the cumulative net variation of plasma amino acid enrichments derived from the intravenous and orally administered tracer L-[*ring*-^13^C_6_]-phenylalanine over the 6 h post-prandial period. E represents the mean plasma amino acid enrichment derived from the intravenous and orally administered tracer L-[*ring*-^13^C_6_]-phenylalanine over the 6 h post-prandial period. EXO­_pro_ represents the exogenous cumulative plasma bioavailability of dietary protein-derived phenylalanine over the 6 h post-prandial period. ΔC represents the cumulative net variation of plasma phenylalanine concentration over the 6 h post-prandial period.

Whole-body protein synthesis, breakdown, and oxidation rate as well as whole-body net balance over the 6 h post-prandial period were assessed based on plasma phenylalanine and tyrosine kinetics as follows (32, 36).

$$Protein oxidation={total appearance}_{tyr}\cdot\frac{E_{tyr}}{E_{phe}}\cdot\frac{total disappearance}{F_{phe,iv}+{EXO}_{oral tracer}+total disappearance}$$

$$Protein synthesis=total disappearance-oxidation$$

$$Net balance=protein synthesis-protein breakdown$$

Total appearance_tyr_ represents the total tyrosine appearance over the 6 h post-prandial period based on L-[*ring*-3,5-^2^H_2_]-tyrosine infusion and plasma enrichments. E_tyr_ represents the mean plasma L-[*ring*-^13^C_6_]-tyrosine enrichment over the 6 h post-prandial period. E_phe_ represents the mean plasma L-[*ring-*^13^C_6_]-phenylalanine enrichment over the 6 h post-prandial period. The calculated whole-body protein synthesis, breakdown, and oxidation rate as well as whole-body net balance over the 6 h post-prandial period (µmol·kg BM^-1^·360 min^-1­­^) were converted to an average rate over the 6 h post-prandial period (µmol·kg BM^-1^·min^-1^).

**SUPPLEMENTAL FIGURE 1.**

Participant flow chart according CONSORT flow diagram for parallel design. HIGH-GLYC, milk protein with a high glycation level; LOW-GLYC, milk protein with a low glycation level; PLA, placebo.


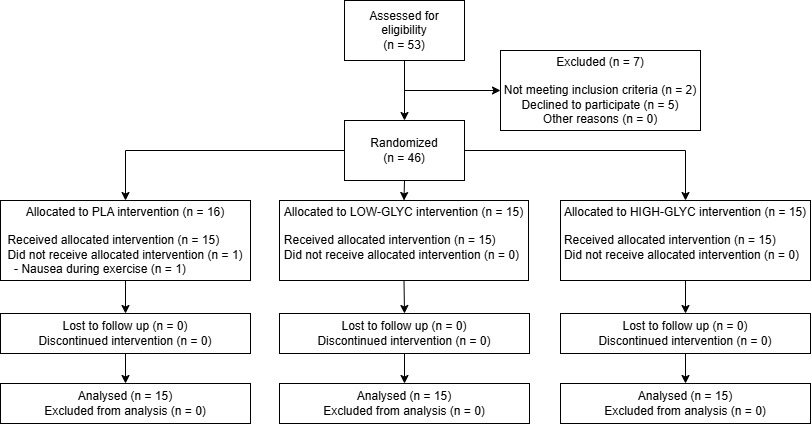


**SUPPLEMENTAL FIGURE 2.**

Plasma non-essential amino acid concentrations, branched-chain amino acid concentrations and individual amino acid concentrations following ingestion of LOW-GLYC, HIGH-GLYC and PLA. Data are analyzed by two-factor repeated measures ANOVA with time as within-subject factor and treatment as between-subject factor. In case of significant interactions, separate one-way ANOVAs were performed for each time period to detect differences between treatments. Bonferroni post hoc test was applied to locate group differences. * indicates LOW-GLYC significantly different from PLA. $ indicates HIGH-GLYC significantly different from PLA. # indicates LOW-GLYC significantly different from HIGH-GLYC. Data are expressed as mean ± standard deviation. BCAA, branched-chain amino acids; HIGH-GLYC, milk protein with a high glycation level; LOW-GLYC, milk protein with a low glycation level; NEAA, non-essential amino acids; PLA, placebo.


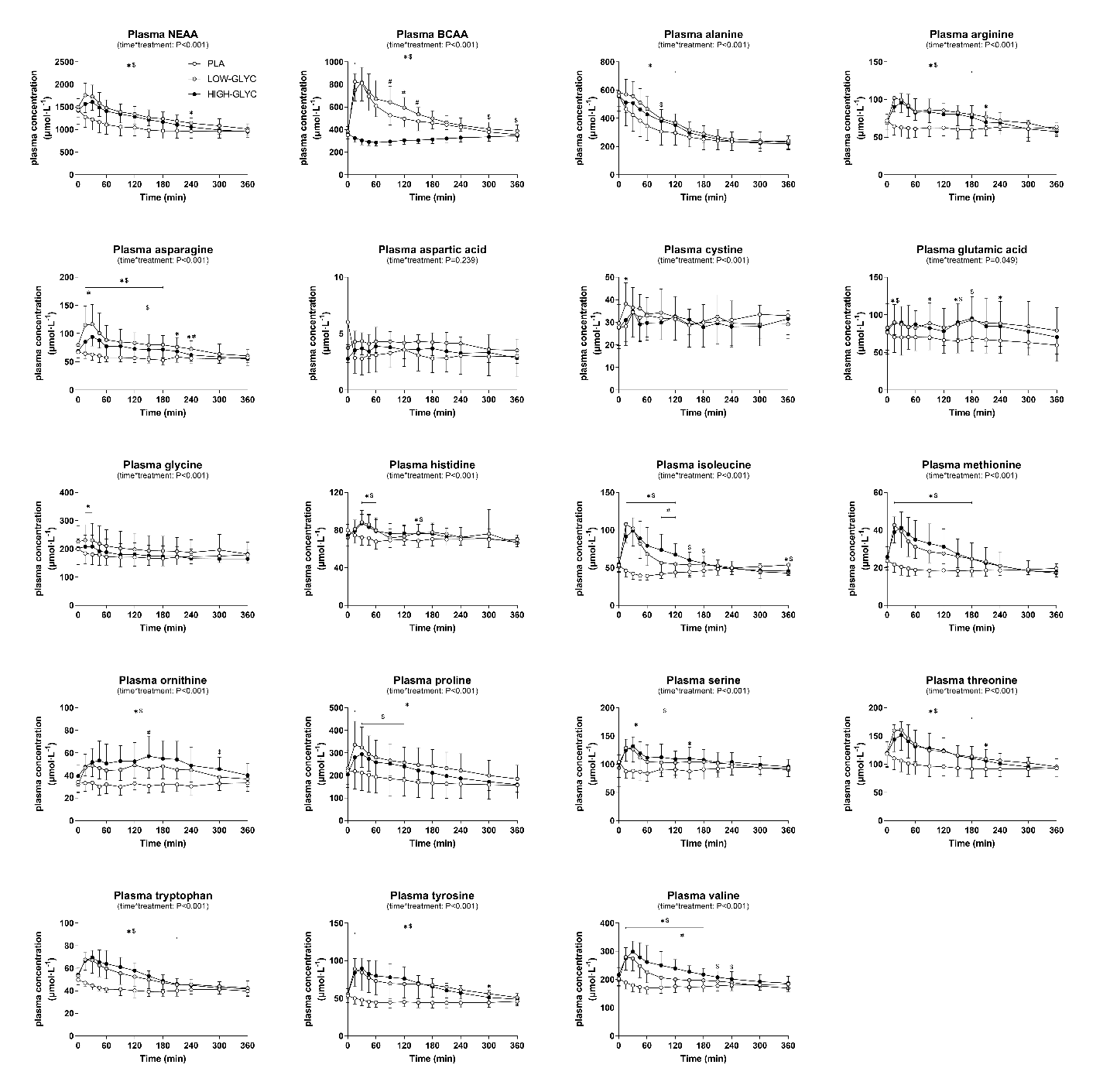

Supplement: Multimedia component 1 [file mmc1.docx]
